# Rivaroxaban Monotherapy in Atrial Fibrillation and Stable Coronary Artery Disease Across Body Mass Index Categories

**DOI:** 10.1016/j.jacasi.2022.08.004

**Published:** 2022-08-27

**Authors:** Masanobu Ishii, Koichi Kaikita, Satoshi Yasuda, Masaharu Akao, Junya Ako, Tetsuya Matoba, Masato Nakamura, Katsumi Miyauchi, Nobuhisa Hagiwara, Kazuo Kimura, Atsushi Hirayama, Kunihiko Matsui, Hisao Ogawa, Kenichi Tsujita, AFIRE Investigators

**Affiliations:** a Department of Cardiovascular Medicine, Graduate School of Medical Sciences, Kumamoto University, Kumamoto, Kumamoto, Japan; b Division of Cardiovascular Medicine and Nephrology, Department of Internal Medicine, Faculty of Medicine, University of Miyazaki, Kiyotake, Miyazaki, Japan; c Department of Cardiovascular Medicine, Tohoku University Graduate School of Medicine, Sendai, Miyagi, Japan; d Department of Cardiology, National Hospital Organization Kyoto Medical Center, Kyoto, Kyoto, Japan; e Department of Cardiovascular Medicine, Kitasato University School of Medicine, Sagamihara, Kanagawa, Japan; f Department of Cardiovascular Medicine, Faculty of Medical Sciences, Kyushu University, Fukuoka, Fukuoka, Japan; g Division of Cardiovascular Medicine, Toho University Ohashi Medical Center, Meguro-ku, Tokyo, Japan; h Department of Cardiovascular Medicine, Juntendo Tokyo Koto Geriatric Medical Center, Koto-ku, Tokyo, Japan; i Department of Cardiology, Tokyo Women's Medical University, Shinjuku-ku, Tokyo, Japan; j Cardiovascular Center, Yokohama City University Medical Center, Yokohama, Kanagawa, Japan; k Department of Cardiology, Osaka Police Hospital, Osaka, Osaka, Japan; l Department of General Medicine and Primary Care, Kumamoto University Hospital, Kumamoto, Kumamoto, Japan; m Kumamoto University, Kumamoto, Kumamoto, Japan

**Keywords:** atrial fibrillation, coronary artery disease, obese, underweight, AF, atrial fibrillation, BMI, body mass index, CAD, coronary artery disease, DOAC, direct oral anticoagulants, MACCE, major adverse cardiac and cerebral event(s), NACE, net adverse clinical event(s)

## Abstract

**Background:**

The AFIRE (Atrial Fibrillation and Ischemic Events With Rivaroxaban in Patients With Stable Coronary Artery Disease) trial showed both noninferiority for efficacy and superiority for safety endpoints of rivaroxaban monotherapy compared with those of rivaroxaban plus antiplatelet therapy (combination therapy) in patients with atrial fibrillation and stable coronary artery disease.

**Objectives:**

This study sought to evaluate outcomes of rivaroxaban monotherapy in those patients across body mass index (BMI) categories.

**Methods:**

Patients were categorized into 4 groups: underweight (BMI <18.5 kg/m^2^), normal weight (BMI 18.5 to <25 kg/m^2^), overweight (BMI 25 to <30 kg/m^2^), and obesity (BMI ≥30 kg/m^2^). Efficacy (a composite of all-cause death, myocardial infarction, unstable angina requiring revascularization, stroke, or systemic embolism) and safety (major bleeding defined according to International Society on Thrombosis and Haemostasis criteria) were compared between rivaroxaban monotherapy and combination therapy across BMI categories.

**Results:**

This study analyzed 2,054 patients with a median age of 75.0 years and CHA_2_DS_2_-VASc score of 4. A significant interaction was not observed between BMI categories and effect of monotherapy for efficacy (*P =* 0.83) and safety (*P =* 0.07), although monotherapy was superior to combination therapy for efficacy in normal weight (HR: 0.64; 95% CI: 0.44-0.95) and safety in overweight (HR: 0.25; 95% CI: 0.10-0.62), whereas a significant difference in the endpoints was not observed in the other BMI categories.

**Conclusions:**

Rivaroxaban monotherapy had a similar effect on prognosis across all BMI categories in patients with atrial fibrillation and stable coronary artery disease. (Atrial Fibrillation and Ischemic Events With Rivaroxaban in Patients With Stable Coronary Artery Disease [AFIRE]; UMIN000016612, NCT02642419)

The AFIRE (Atrial Fibrillation and Ischemic Events With Rivaroxaban in Patients With Stable Coronary Artery Disease) trial demonstrated that rivaroxaban monotherapy was noninferior for cardiovascular events or death from any cause and was superior for major bleeding events to rivaroxaban plus antiplatelet therapy (combination therapy) in patients with atrial fibrillation (AF) and stable coronary artery disease (CAD) over 1 year after revascularization or those with angiographically confirmed CAD not requiring revascularization.^1^ This result provides fundamental evidence of guideline-recommended antithrombotic management with direct oral anticoagulant (DOAC) monotherapy for patients with AF and stable CAD.^2^

In the AFIRE trial, the patients received fixed-dose rivaroxaban according to their creatinine clearance, irrespective of body weight or body mass index (BMI).^3^ However, a previous pharmacokinetic study showed that about 20% of patients who are obese (over 120 kg) had a peak plasma concentration of DOACs below the therapeutic range of peak concentration, suggesting potential risk for underdosing of DOACs in patients who are extremely obese.^4^ In current guidelines, avoiding use of DOACs has been recommended in patients with BMI >40 kg/m^2^ or weight >120 kg.^5^^,^^6^ In addition to the caution in extreme obesity, the European Heart Rhythm Association mentioned high bleeding risk with anticoagulation therapy in patients who are severely underweight.^6^ The guidelines also proposed checking for accumulation of the drug when using DOACs for patients with extreme BMI.^5^^,^^6^

Patients with AF and stable CAD have high risk of thrombotic cardiovascular and cerebrovascular events, as well as bleeding events, thus, the high risk of antithrombotic therapy-related bleeding complications remains a problem in those patients with extreme BMI. However, no accumulating evidence regarding efficacy and safety of these fixed-dose DOAC monotherapy or combination with antiplatelet drugs has been established in this population.

The aim of this post hoc analysis of the AFIRE trial was to evaluate outcomes of rivaroxaban monotherapy (vs combination therapy) in patients with AF and stable CAD across BMI categories.

## Methods

### Study design and study participants

This subanalysis of the AFIRE trial was a post hoc analysis. The detailed study design, protocol, and the primary results of the AFIRE trial have been published elsewhere.^1^^,^^3^ Briefly, the AFIRE trial was a multicenter, randomized, open-label, parallel-group trial that was performed at 294 Japanese institutions between February 23, 2015, and September 30, 2017. The patients aged ≥20 years who were Japanese and diagnosed with AF with a CHADS_2_ score ≥1 and stable CAD at ≥1 year after revascularization or those with angiographically confirmed CAD not requiring revascularization were enrolled. Exclusion criteria was as follows: a history of stent thrombosis; coexisting active cancer; or poorly controlled hypertension. Patients were allocated in a 1:1 ratio to receive either rivaroxaban (10 mg once daily for patients with a creatinine clearance rate of 15 to 49 mL/min or 15 mg once daily for patients with a creatinine clearance rate ≥50 mL/min) alone or rivaroxaban plus an antiplatelet drug (either aspirin or P2Y_12_ inhibitor). After exclusion of patients with missing value of BMI, the patients were divided into 4 groups; underweight (BMI <18.5 kg/m^2^), normal weight (BMI 18.5 to <25 kg/m^2^), overweight (BMI 25 to <30 kg/m^2^), and obesity (BMI ≥30 kg/m^2^). The patient follow-up period was at least 24 months and up to 45 months after randomization.

All patients provided written informed consent. The trial was performed in accordance with the Declaration of Helsinki and approved by the Institutional Review Boards of the National Cerebral and Cardiovascular Center and all participating institutions. An independent data and safety monitoring committee reviewed the data collected throughout the trial.

### Study endpoints

The primary efficacy endpoint of this study was defined as a composite of all-cause death, myocardial infarction, unstable angina requiring revascularization, stroke, or systemic embolism. The primary safety endpoint was defined as a major bleeding event according to the International Society on Thrombosis and Haemostasis criteria.^7^ Net adverse clinical event (NACE) was defined as a composite of all-cause death, myocardial infarction, stroke, or major bleeding according to the criteria of the International Society on Thrombosis and Haemostasis. Major adverse cardiac and cerebral event (MACCE) was defined as a composite of cardiac death, myocardial infarction, unstable angina requiring revascularization, stroke, or systemic embolism.

### Data availability

The data underlying this paper will be shared on reasonable request to the corresponding author.

### Statistical analysis

Data are presented as the median (IQR) for continuous variables and number (percentage) for categorical variables. Group comparisons were performed by Kruskal-Wallis test for continuous variables, the chi-square test or Fisher exact test for categorical variables, and the log-rank test for Kaplan-Meier curves, as appropriate. Cox proportional hazard regression was performed to compute HRs and 95% CIs as estimates for the study endpoints. The interaction test was performed to assess the effect modification by BMI categories. Event rates (per person-years) and the 95% CIs for both rivaroxaban monotherapy and combination therapy were estimated using linear model with a natural regression spline with a degree-of-freedom of 2, which set 2 boundary knots and 1 internal knot, placed at the 50th quantile of BMI. To assess the robustness of the efficacy and safety of monotherapy across BMI categories, the sensitivity analyses were performed with respect to other BMI categories using cutoff of quintiles of BMI. Two-sided *P* < 0.05 was statistically significant. All statistical analyses were performed using SPSS (version 23.0, IBM Corp) and R software (version 4.0.5, R Foundation).

## Results

### Study participants

**Figure 1:**
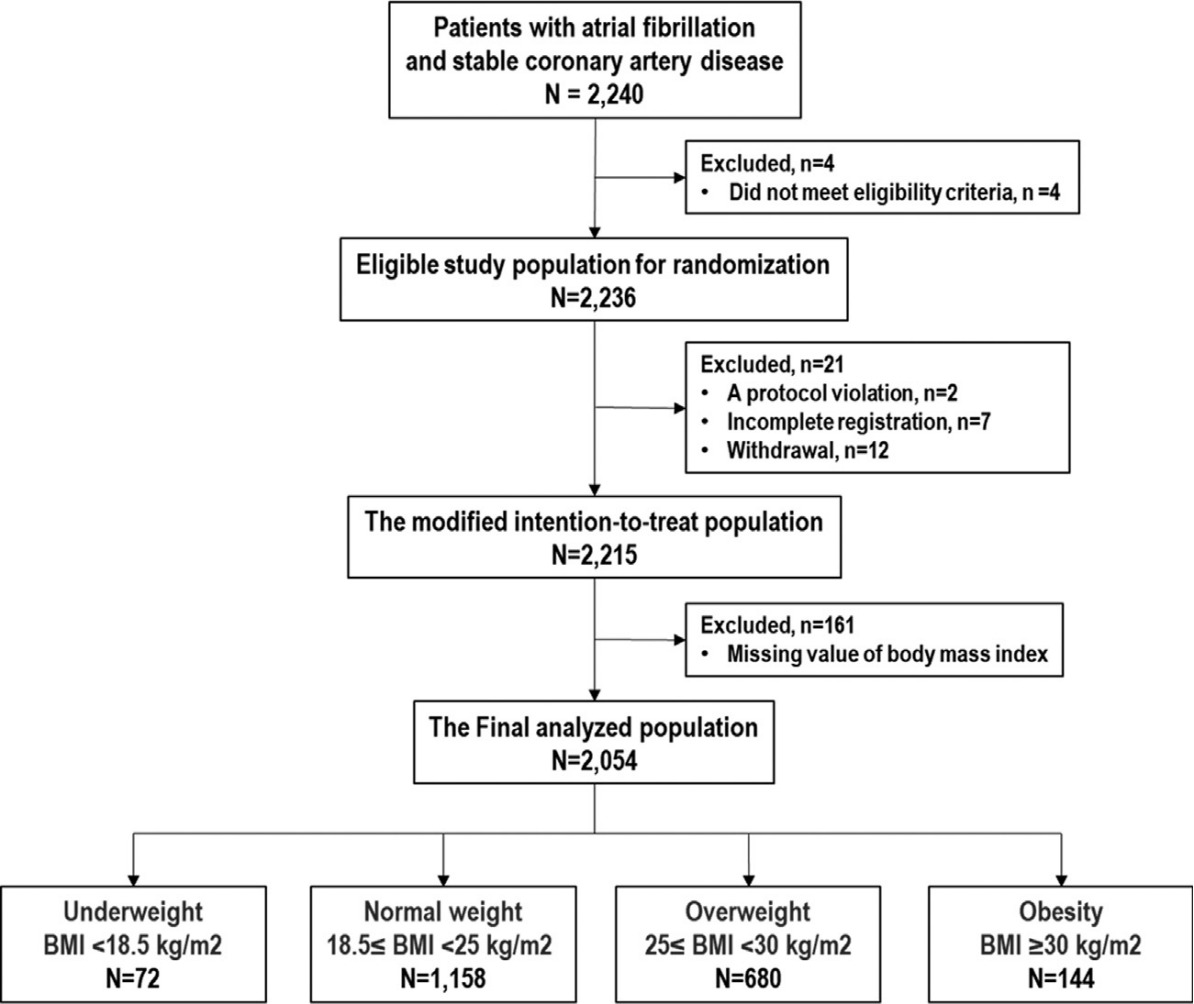
Study Flowchart Flowchart of study population enrolled in the subanalysis. BMI = body mass index.

In this post hoc analysis, 2,054 patients were analyzed after exclusion of 161 patients (7%) with missing value of BMI from 2,215 patients in the modified intention-to-treat population of the AFIRE trial (Figure 1). Overall, patients with a median age of 75.0 (IQR: 69-80) years and CHA_2_DS_2_-VASc score of 4 (IQR: 3-5) were predominantly male (79%). Baseline characteristics of the study population categorized by BMI are shown in Table 1. Of all patients, 72 (3.5%) were underweight (BMI <18.5 kg/m^2^), 1,158 (56.4%) were normal weight (BMI 18.5 to <25 kg/m^2^), 680 (33.1%) were overweight (BMI 25 to <30 kg/m^2^), and 144 (7.0%) were obese (BMI ≥30 kg/m^2^). The median ages, respectively, were 78.5 (IQR: 72.3-83.0), 76.0 (IQR: 71.0-81.0), 73.0 (IQR: 68.0-79.0), and 69.5 (IQR: 61.0-76.8) years, and the median BMIs, respectively, were 17.7 (IQR: 16.8-18.1), 22.7 (IQR: 21.2-23.8), 26.8 (IQR: 25.8-27.9), and 32.2 (IQR: 30.9-33.7) kg/m^2^ (*P <* 0.001 for both). The median creatinine clearances were 40.7 (IQR: 30.9-51.6), 54.1 (IQR: 41.7-66.6), 67.3 (IQR: 53.3-83.6), and 86.0 (IQR: 67.0-118.0) mL/min and 62%, 52%, 36%, and 30% of patients received a reduced dose of rivaroxaban in the underweight through obesity groups, respectively (*P <* 0.001 for both). There was no statistical difference in allocation (rivaroxaban monotherapy or combination therapy), the mean CHA_2_DS_2_-VASc, and high score (≥3) of HAS-BLED score. In the 1,022 patients with the combination therapy, the details of antiplatelet agents were as follows: low-dose aspirin 81-100 mg/d (n = 738, 72%); regular dose of clopidogrel (n = 244, 24%); reduced dose of clopidogrel (n = 19, 1.9%); regular dose of prasugrel (n = 16, 1.6%); reduced dose of prasugrel (n = 1, 0.1%); and ticlopidine (n = 2, 0.2%).

**Table 1:** Baseline Characteristics of the Study Population Across BMI Categories

	Overall (N = 2,054)	BMI Categories^a^				*P* Value
		Underweight (n = 72)	Normal Weight (n = 1,158)	Overweight (n = 680)	Obesity (n = 144)	
Intervention						0.89
Rivaroxaban monotherapy	1,032 (50)	33 (46)	580 (50)	346 (51)	73 (51)	
Combination therapy	1,022 (50)	39 (54)	578 (50)	334 (49)	71 (49)	
Reduced dose of rivaroxaban	932 (46)	43 (62)	602 (52)	244 (36)	43 (30)	<0.001
Age, y	75.0 (69.0-80.0)	78.5 (72.3-83.0)	76.0 (71.0-81.0)	73.0 (68.0-79.0)	69.5 (61.0-76.8)	<0.001
Male	1,628 (79)	43 (60)	916 (79)	564 (83)	105 (73)	<0.001
Weight, kg	63.7 (56.0-72.0)	43.5 (40.4-48.2)	59.0 (53.0-64.5)	72.0 (66.0-77.7)	87.3 (78.0-95.0)	<0.001
BMI, kg/m^2^	24.2 (22.0-26.6)	17.7 (16.8-18.1)	22.7 (21.2-23.8)	26.8 (25.8-27.9)	32.2 (30.9-33.7)	<0.001
Current smoker	275 (13)	7 (9.7)	157 (14)	91 (13)	20 (14)	0.83
Hypertension	1,762 (86)	58 (81)	966 (83)	602 (89)	136 (94)	<0.001
Diabetes	863 (42)	22 (31)	427 (37)	322 (47)	92 (64)	<0.001
Dyslipidemia	1,428 (70)	40 (56)	777 (67)	492 (72)	119 (83)	<0.001
Previous stroke	295 (14)	11 (15)	181 (16)	85 (12)	18 (12)	0.28
Previous myocardial infarction	716 (35)	20 (28)	399 (34)	234 (34)	63 (44)	0.081
Previous PCI	1,455 (71)	42 (58)	824 (71)	482 (71)	107 (74)	0.096
Type of stent						0.68
BMS	314 (23)	6 (16)	182 (24)	105 (24)	21 (22)	
DES	918 (68)	26 (68)	517 (68)	306 (69)	69 (71)	
Both types	52 (3.9)	4 (11)	31 (4.1)	14 (3.2)	3 (3.1)	
Unknown	59 (4.4)	2 (5.3)	34 (4.5)	19 (4.3)	4 (4.1)	
Previous CABG	233 (11)	13 (18)	139 (12)	70 (10)	11 (7.6)	0.085
Type of atrial fibrillation						<0.001
Paroxysmal	1,083 (53)	52 (72)	636 (55)	339 (50)	56 (39)	
Persistent	317 (15)	6 (8.3)	160 (14)	119 (18)	32 (22)	
Permanent	654 (32)	14 (19)	362 (31)	222 (33)	56 (39)	
Creatinine clearance, mL/min	59.2 (45.2-74.8)	40.7 (30.9-51.6)	54.1 (41.7-66.6)	67.3 (53.3-83.6)	86.0 (67.0-118.0)	<0.001
Missing value	16	2	8	5	1	
Creatinine category						
<30 mL/min	111 (5.4)	16 (23)	79 (6.9)	14 (2.1)	2 (1.4)	
30 to <50 mL/min	570 (28)	34 (49)	404 (35)	121 (18)	11 (7.7)	
≥50 mL/min	1,357 (67)	20 (29)	667 (58)	540 (80)	130 (91)	
CHADS_2_ score	2 (2, 3)	2 (2, 3)	2 (2, 3)	2 (2, 3)	2 (2, 3)	0.22
CHA_2_DS_2_-VASc score	4 (3, 5)	4 (3, 5)	4 (3, 5)	4 (3, 5)	4 (3, 5)	0.10
HAS-BLED score	2 (2, 3)	2 (2, 2.75)	2 (2, 3)	2 (2, 3)	2 (1, 2)	0.031
≥3	535 (27)	18 (25)	312 (28)	175 (26)	30 (21)	0.40
Missing value	53	0	34	16	3	

Values are n, n (%), or median (IQR).

BMI = body mass index; BMS = bare metal stent(s); CABG = coronary artery bypass graft; DES = drug-eluting stent(s); PCI = percutaneous coronary intervention.

a Underweight: BMI <18.5 kg/m^2^; normal weight: 18.5 kg/m^2^ to <25.0 kg/m^2^; overweight: 25.0 kg/m^2^ to <30.0 kg/m^2^; obesity: BMI ≥30.0 kg/m^2^.

### Association of BMI on study endpoints

The median follow-up period was 721 (IQR: 505, 941) days. During follow-up, 201 events (9.8%) of the primary efficacy endpoint, 87 (4.2%) of the primary safety endpoint, 203 (9.9%) of NACE, and 162 (7.9%) of MACCE were observed. Among the total population, compared with the normal weight, the underweight group was associated with a significantly higher risk of primary efficacy endpoint (HR: 2.22; 95% CI: 1.25-3.93; *P =* 0.006), whereas overweight and obesity groups were not (Table 2). This association was also observed in the rivaroxaban monotherapy, but not in the combination therapy. For the primary safety endpoint, difference in BMI categories was not associated with the high risk. In the monotherapy, the overweight group was associated with a lower risk (HR: 0.36; 95% CI: 0.13-0.98), compared with the normal weight group. For the NACE and MACCE incidence, the underweight group was associated with a significantly higher risk (HR: 2.38; 95% CI: 1.39-4.08; and HR: 2.11; 95% CI: 1.11-4.01, respectively), compared with the normal weight group, whereas the overweight and obesity groups were not (Table 2). These tendencies were observed in the monotherapy, but not in the combination therapy.

**Table 2:** Risks of Study Endpoints in Underweight and Excess BMI Categories Compared With Normal BMI

	Total Population (N = 2,054)				Rivaroxaban Monotherapy (n = 1,032)		Combination Therapy (n = 1,022)	
	Subgroup	Events	HR (95% CI)	*P* Value	HR (95% CI)	*P* Value	HR (95% CI)	*P* Value
Primary efficacy endpoint		201 (9.8)						
Underweight^a^	72	15 (20.8)	2.21 (1.25-3.92)	0.006	3.86 (1.75-8.48)	0.001	1.39 (0.59-3.29)	0.46
Normal weight	1,158	108 (9.3)	Reference	Reference	Reference	Reference	Reference	Reference
Overweight	680	64 (9.4)	1.26 (0.91-1.75)	0.16	1.54 (0.93-2.53)	0.091	1.15 (0.74-1.78)	0.55
Obesity	144	14 (9.7)	1.67 (0.91-3.08)	0.099	1.61 (0.58-4.47)	0.36	1.77 (0.82-3.82)	0.15
Primary safety endpoint		87 (4.2)						
Underweight	72	3 (4.2)	0.85 (0.26-2.77)	0.79	N/A	N/A	1.56 (0.45-5.35)	0.48
Normal weight	1,158	54 (4.7)	Reference	Reference	Reference	Reference	Reference	Reference
Overweight	680	27 (4.0)	0.96 (0.58-1.57)	0.86	0.36 (0.13-0.98)	0.045	1.45 (0.79-2.65)	0.23
Obesity	144	3 (2.1)	0.73 (0.21-2.51)	0.62	1.20 (0.24-6.15)	0.82	0.49 (0.06-3.82)	0.49
Net adverse clinical events		203 (9.9)						
Underweight	72	17 (23.6)	2.38 (1.39-4.08)	0.002	3.04 (1.38-6.70)	0.006	1.75 (0.82-3.75)	0.15
Normal weight	1,158	115 (9.9)	Reference	Reference	Reference	Reference	Reference	Reference
Overweight	680	57 (8.4)	0.98 (0.70-1.37)	0.90	0.86 (0.49-1.51)	0.60	1.05 (0.68-1.60)	0.84
Obesity	144	14 (9.7)	1.48 (0.80-2.72)	0.21	1.60 (0.57-4.50)	0.37	1.56 (0.72-3.34)	0.26
Major adverse cardiac and cerebral events		162 (7.9)						
Underweight	72	12 (16.7)	2.11 (1.11-4.01)	0.023	2.71 (1.03-7.10)	0.043	1.93 (0.80-4.64)	0.14
Normal weight	1,158	90 (7.8)	Reference	Reference	Reference	Reference	Reference	Reference
Overweight	680	50 (7.4)	1.16 (0.81-1.68)	0.42	1.62 (0.95-2.76)	0.078	0.96 (0.57-1.60)	0.87
Obesity	144	10 (6.9)	1.29 (0.64-2.63)	0.48	0.98 (0.28-3.48)	0.98	1.50 (0.63-3.56)	0.36

Values are n and n (%) for subjects and events, respectively. HRs with 95% CIs were adjusted for age, sex, allocation, dose of rivaroxaban, hypertension, diabetes, dyslipidemia, prior myocardial infarction, revascularization, type of atrial fibrillation, creatinine clearance, and CHA_2_DS_2_-VASc score.

N/A = not applicable; other abbreviations as in Table 1.

a Underweight: BMI <18.5 kg/m^2^; normal weight: 18.5 kg/m^2^ to <25.0 kg/m^2^; overweight: 25.0 kg/m^2^ to <30.0 kg/m^2^; obesity: BMI ≥30.0 kg/m^2^.

### Study endpoints of rivaroxaban monotherapy versus combination therapy across BMI categories

**Figure 2:**
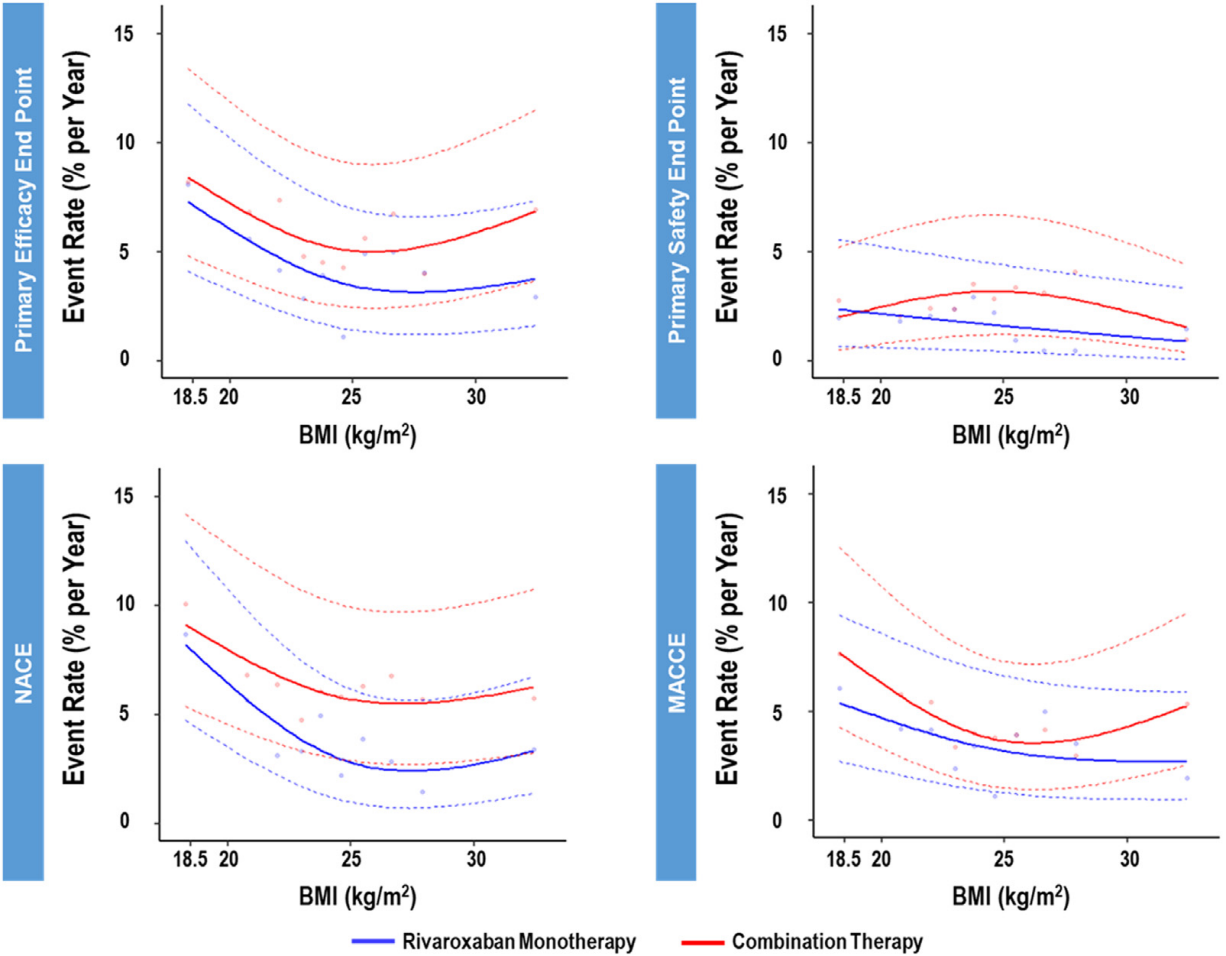
Event Rates in Monotherapy Versus Combination Therapy According to BMI The **lines** show incidence rates of clinical outcomes (primary efficacy, primary safety, net adverse clinical event, and major adverse cardiovascular event) between rivaroxaban monotherapy and combination therapy according to body mass index (BMI) of participants. The **solid circles** indicate incidence rates for groups divided by 10 quantiles of BMI in each arm. MACCE = major adverse cardiac and cerebral event(s); NACE = net adverse clinical event(s).

As shown in Figure 2, in event rates for the primary efficacy endpoint, NACE, and MACCE, yearly incidence tended to be lower in rivaroxaban monotherapy than those in combination therapy, with reverse-J-shape curve in the relationship between the incidence and BMI. On the other hand, the relationship was not evident in the rate of primary safety endpoint.

**Figure 3:**
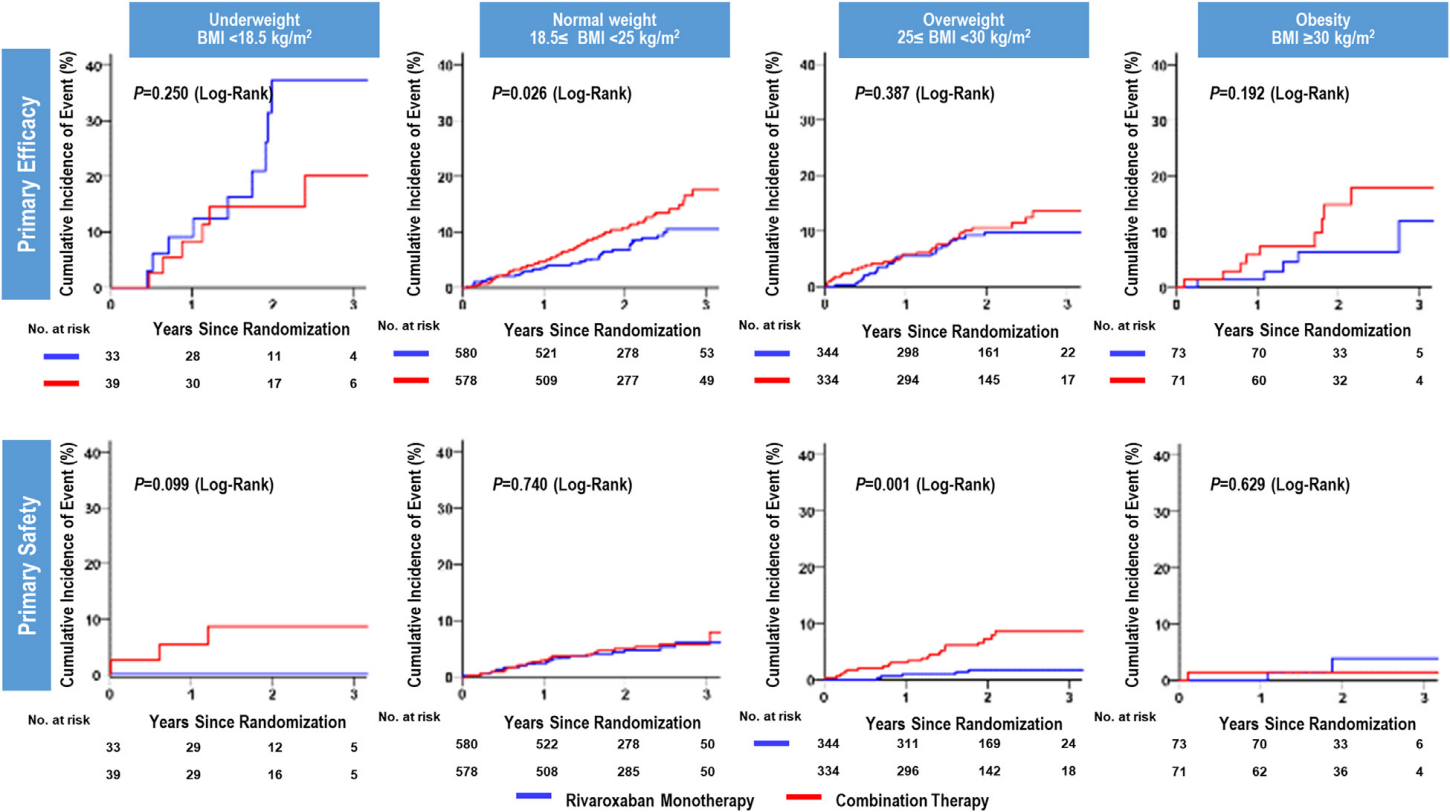
Primary Endpoint in Monotherapy Versus Combination According to BMI Patients with atrial fibrillation and stable coronary artery disease are stratified according to the body mass index (BMI) categories. At 3 years, the rate of primary efficacy endpoint was significantly lower in patients with monotherapy than in those with combination therapy in normal BMI, and the rate of primary safety endpoint was significantly lower in patients with monotherapy than in those with combination therapy in the overweight group.

**Figure 4:**
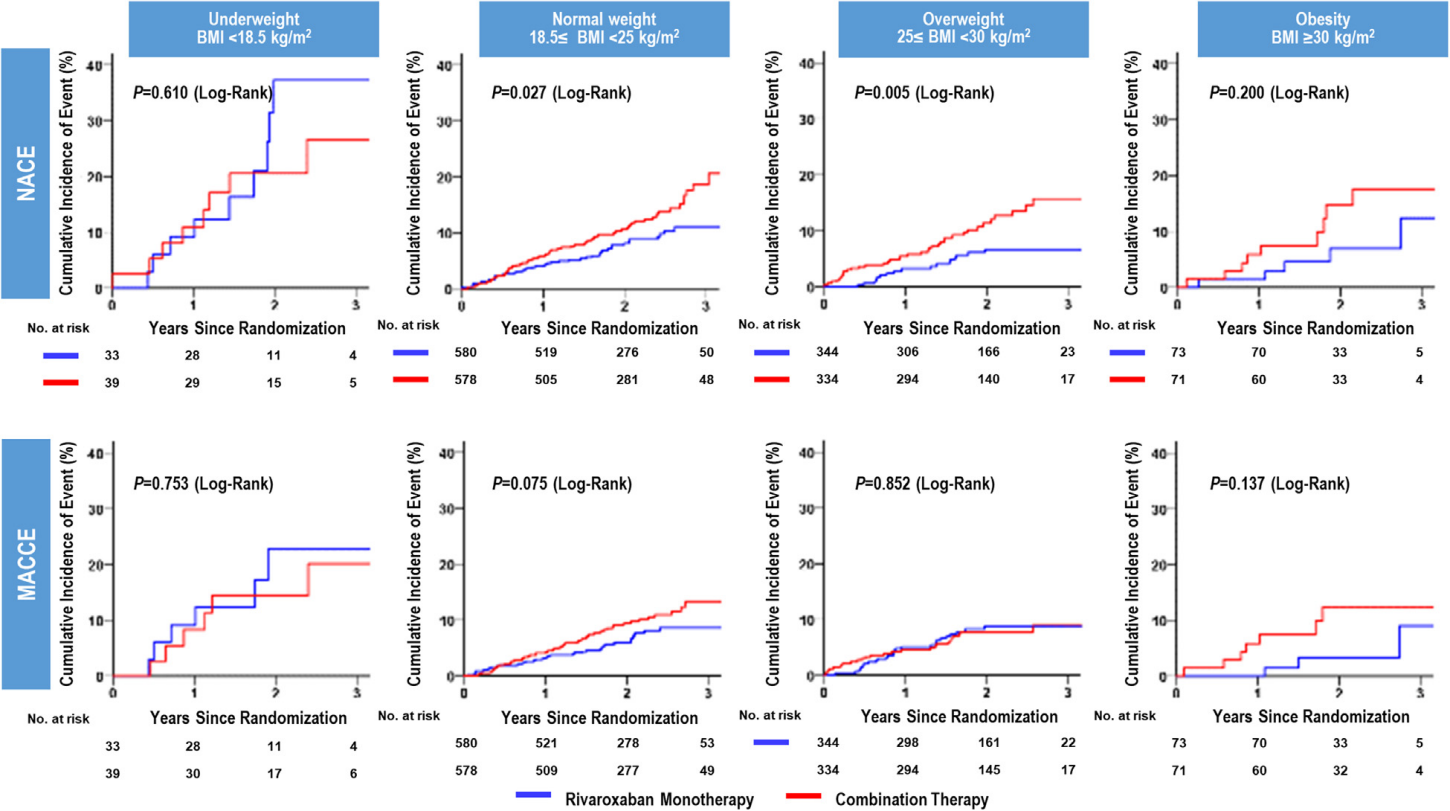
NACE and MACCE in Monotherapy Versus Combination Therapy According to BMI Patients with atrial fibrillation and stable coronary artery disease are stratified according to the BMI categories. At 3 years, the rate of NACE was significantly lower in patients with monotherapy than in those with combination therapy in normal and overweight BMI groups. Abbreviations as in Figure 2.

Kaplan-Meier curves estimated lower cumulative incidence of primary endpoint in rivaroxaban monotherapy than those in combination therapy in normal weight group (*P =* 0.026), and lower cumulative incidence of primary safety endpoint in rivaroxaban monotherapy than those in combination therapy in overweight group (*P =* 0.001) (Figure 3). For NACE, the cumulative incidence was reduced by rivaroxaban monotherapy in normal weight and overweight groups (*P =* 0.027; *P =* 0.005, respectively) (Figure 4).

**Figure 5:**
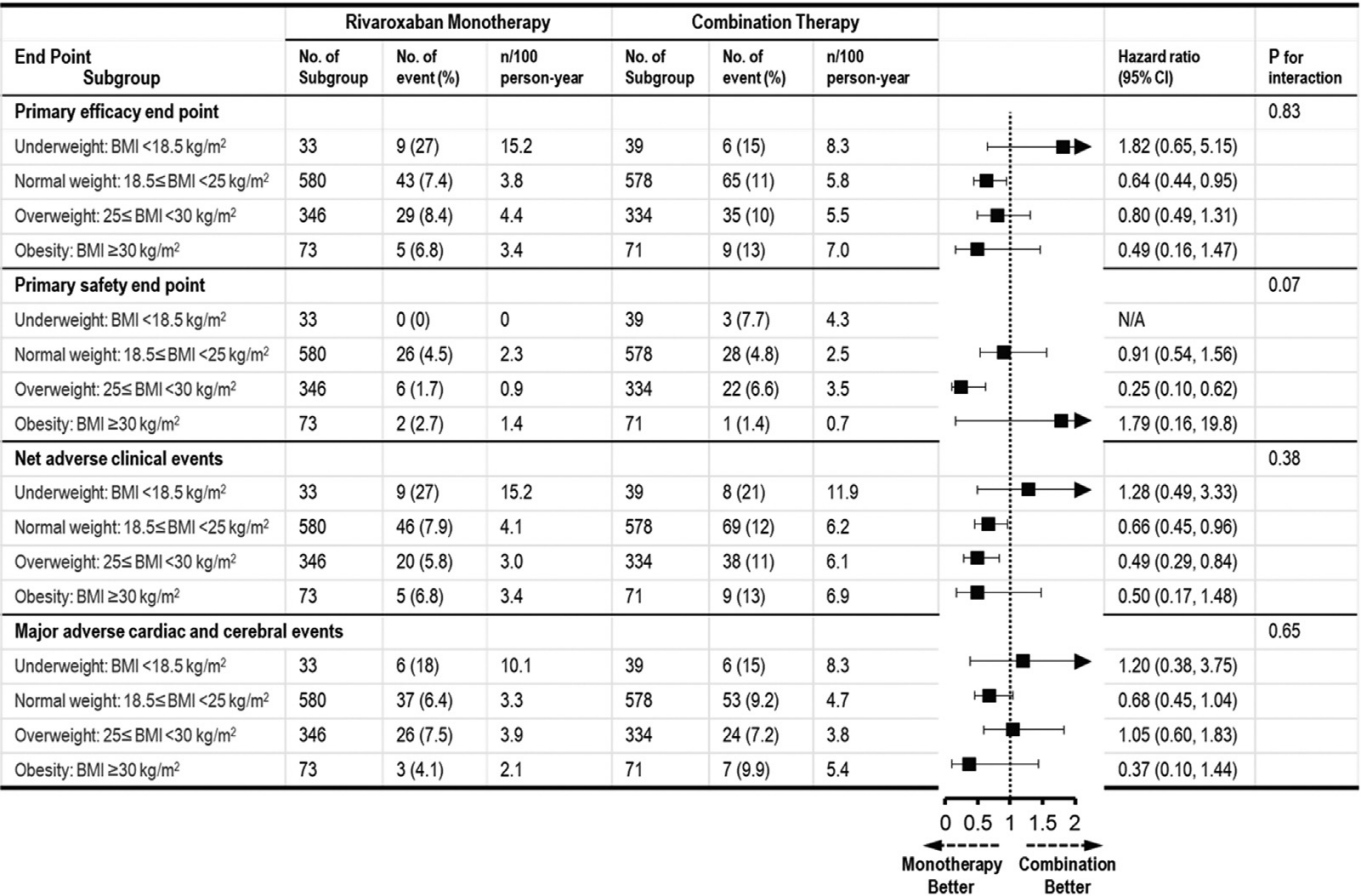
Study Endpoints of Rivaroxaban Monotherapy in Relation to BMI Categories Crude HRs and 95% CIs for the study endpoints were estimated using the Cox proportional hazard regression model. Testing interaction between the treatment and the subgroup was performed. BMI = body mass index; N/A = not available.

Although the sample sizes for underweight and obesity groups were limited, monotherapy was superior to combination therapy for primary efficacy in normal weight group (HR: 0.64; 95% CI: 0.44-0.95), primary safety in overweight group (HR: 0.25; 95% CI: 0.10-0.62), and NACE in normal weight group (HR: 0.66; 95% CI: 0.45-0.96) and overweight group (HR: 0.49; 95% CI: 0.29-0.84), whereas a significant difference in the endpoints was not observed in the other BMI categories (Figure 5). Significant interaction was not observed in the relationship between BMI and the effect of rivaroxaban monotherapy on study endpoints.

**Figure 6:**
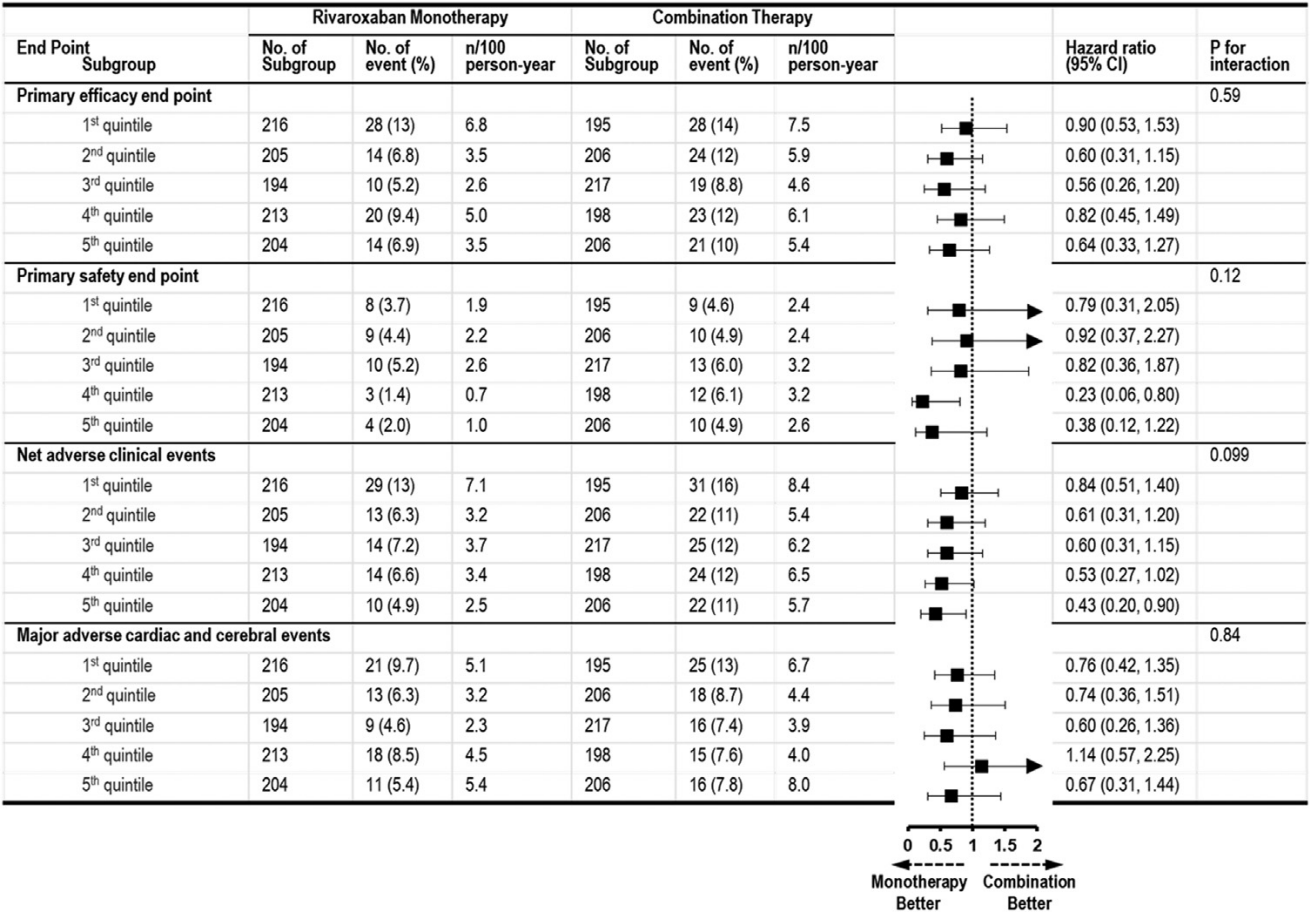
Sensitivity Analysis of Study Endpoints Sensitivity analysis using alternative cutoff of body mass index (quintile) was performed to compute crude HRs and 95% CIs for the study endpoints in the Cox proportional hazard regression model. Testing interaction between the treatment and the subgroup was performed.

The results of sensitivity analysis using other BMI categories with cutoff of quintiles of BMI were almost consistent with the primary analysis results (Figure 6). Monotherapy was superior to combination therapy for primary safety in fourth quintile of BMI (HR: 0.23; 95% CI: 0.06-0.80) and NACE in fifth quintile (HR: 0.43; 95% CI: 0.20-0.90), whereas a significant difference in the endpoints was not observed in the other BMI categories, with no significant interaction.

## Discussion

The main findings of this subanalysis of the AFIRE trial were as follows: 1) patients with lower BMI were the older, had the lower creatinine clearance, and had the higher prevalence of reduced dose; 2) rivaroxaban monotherapy was superior to combination therapy for primary efficacy in normal BMI group, primary safety in overweight group, and NACE in normal BMI and overweight groups, whereas a significant difference in the endpoints was not observed in the other BMI categories although the underweight and obese populations had small sample sizes; and 3) significant interaction was not observed in the relationship between BMI and the effect of rivaroxaban monotherapy on study endpoints. Based on these findings, rivaroxaban monotherapy might be a potential therapeutic option with safety and efficacious antithrombotic regimens for patients with stable CAD and AF across a broad range of BMIs (Central Illustration).

**Central Illustration:**
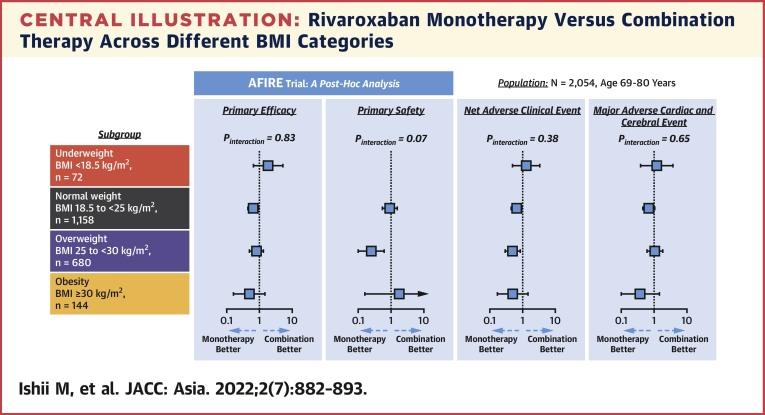
Rivaroxaban Monotherapy Versus Combination Therapy Across Different BMI Categories This post hoc subanalysis of AFIRE (Atrial Fibrillation and Ischemic Events With Rivaroxaban in Patients With Stable Coronary Artery Disease) trial indicated that rivaroxaban monotherapy had similar effect on clinical outcomes across all body mass index (BMI) categories in patients with atrial fibrillation and stable coronary artery disease. MACCE = major adverse cardiac and cerebral event(s); NACE = net adverse clinical event(s).

Although in patients with severe obesity (BMI >40 kg/m^2^ or body weight >120 kg), because of the potential risk of underdosing of DOACs, use of DOACs has not been recommended,^5^^,^^6^ whereas previous studies suggested efficacy and safety of DOACs compared with warfarin in patients with AF irrespective of BMI.^8^^,^^9^ Barakat et al ^8^ conducted a retrospective single-center study analyzing 36,094 consecutive patients with AF and CHA_2_DS_2_-VASc of ≥1 who were receiving anticoagulation. Their study^8^ showed that DOACs compared to warfarin were associated with better efficacy and safety in patients with wide range of BMIs, even in patients who are underweight (BMI <18.5 kg/m^2^) or morbidly obese (BMI >40 kg/m^2^). A meta-analysis also demonstrated that DOACs, compared with warfarin, were associated with reduction in risks of stroke or systemic embolism and major bleeding in patients who are underweight, normal weight, and overweight, and DOACs were not inferior to warfarin in patients who are obese.^9^ However, because only 2 randomized controlled trials—OAC-ALONE (Optimizing Antithrombotic Care in Patients with Atrial Fibrillation and Coronary Stent)^10^ and the AFIRE trial—have investigated effect of oral anticoagulant monotherapy versus combination therapy in patients with AF and stable CAD, effect modification by BMI in association between monotherapy and clinical outcomes has not been fully investigated. Therefore, to the best of our knowledge, this post hoc analysis of the AFIRE trial was the first study to evaluate efficacy and safety of DOAC monotherapy compared with combination of DOACs with antiplatelet agent across all BMI categories. The present study showed that monotherapy, compared with combination therapy, was not significantly associated with increase in MACCE risk regardless of a wide range of BMIs, indicating that clinicians might select DOAC monotherapy rather than combination therapy without a concern of increased risk of thrombotic events in patients with extreme BMI.

The plausible mechanisms of consistency of effect of rivaroxaban monotherapy on clinical outcomes across different BMI categories might be explained by the following 3 factors. First, cardiovascular event tends to be subsequently triggered after a bleeding event. The results of another subanalysis of the AFIRE trial support this fact.^11^ The subanalysis showed that more than 70% of patients suffered both bleeding and subsequently MACCE. In particular, within 30 days after major bleeding, patients had increased MACCE risks (HR: 7.81; 95% CI: 4.20-14.54).^11^ The potential mechanisms for increased cardiovascular events and mortality are assumed to be activation of coagulation cascade, increased prothrombotic cytokines, cessation of antithrombotic therapy, anemia, transfusion, or reflex tachycardia.^12^ Therefore, rivaroxaban monotherapy, which is considered to have a low risk of bleeding events, compared with combination antiplatelet therapy might be associated with no increase in cardiovascular thrombotic events. Second, low BMI or body weight is established as a high bleeding risk for patients undergoing percutaneous coronary intervention with antiplatelet therapy, especially in Japanese populations.^13^, ^14^, ^15^ In the present study, in the BMI <18.5 kg/m^2^ categories, the major bleeding event was observed in 0% of patients (0 of 33) in monotherapy versus in 7.7% (3 of 39) in combination therapy, suggesting that avoiding antiplatelet drug use may have led to prevention of bleeding events. Third, factor Xa inhibitors have pleiotropic effect of inhibition of platelet activation via thrombin-protease–activated receptor pathway in addition to effect of anticoagulation.^16^^,^^17^ In addition, a previous study^18^ using the T-TAS (Total Thrombus-formation Analysis System) revealed that DOACs, compared with the non-anticoagulation therapy, had a significant decrease in PL (Platelet-chip) area under the curve, which indicates suppression of platelet aggregation. In general, being obese or overweight is considered to be a high-risk factor of cardiovascular events,^19^ although there is an obesity paradox.^20^ In any case, the efficacy and safety of DOACs, compared with warfarin, in those patients who are obese with AF has been established. Based on these findings, we therefore speculated that rivaroxaban monotherapy with pleiotropic effect of inhibition of platelet activation may have a sufficient effect of prevention for thrombotic events even in patients with severe obesity.

### Study limitations

First, risk of sampling bias in randomized control trials might affect the results of this study. The study population had few patients with low BMI (underweight) or high BMI (obesity), and the distribution of BMI in this study might be different from that of the real-world setting. The low absolute number of events and study population with extreme BMI categories limits the precision of the estimate of effect of rivaroxaban monotherapy on clinical outcomes. Further studies should be conducted in real-world populations to investigate effect of DOAC monotherapy on clinical outcomes across BMI categories. Second, our study population including only Japanese patients might limit generalizability of the results. In the trial, the Japan-approved rivaroxaban dose of 10 or 15 mg once daily, not the globally approved once-daily dose of 20 mg, was used according to patient’s creatinine clearance. However, previous pharmacokinetics-pharmacodynamics analyses showed rivaroxaban dose of 15 mg once daily in Japanese patients with AF would yield exposures comparable to the dose of 20 mg once daily in White patients with AF.^21^ Third, unmeasured confounding factors might have biased the results of the present study with regard to comparison in each of the BMI categories, because BMI was not included in allocation factors.

## Conclusions

This post hoc subanalysis of the AFIRE trial demonstrated that rivaroxaban monotherapy had similar effect on clinical outcomes across all BMI categories in patients with AF and stable CAD, indicating a fixed-dose DOAC monotherapy might be a safe, applicable antithrombotic regimen in those patients who have a high risk of thrombotic events. Future study is needed to confirm the robustness of the result of this study in real-world populations.

Perspectives **COMPETENCY IN MEDICAL KNOWLEDGE:** The present study showed that a significant interaction was not observed between BMI categories and effect of monotherapy on clinical outcomes, emphasizing that clinicians can select DOAC monotherapy rather than combination therapy regardless of BMI levels in patients with AF and stable CAD.

**TRANSLATIONAL OUTLOOK:** Future study is needed to confirm the consistent effect of rivaroxaban monotherapy on clinical outcomes across different BMI categories in real-world populations.

## Funding Support and Author Disclosures

This work was supported by the Japan Cardiovascular Research Foundation based on a contract with Bayer Yakuhin, Ltd, which did not have a role in the design of the trial, collection or analysis of the data, interpretation of the trial results, or writing of the manuscript. Dr Kaikita has received remuneration for lectures from Bayer Yakuhin, Daiichi Sankyo, Novartis Pharma, and Otsuka Pharmaceutical; has received trust research/joint research funds from Bayer Yakuhin and Daiichi Sankyo; and has received scholarship funds from Abbott Medical. Dr Yasuda has received grants from Takeda Pharmaceutical, Abbott, and Boston Scientific; and personal fees from Daiichi Sankyo and Bristol Myers Squibb. Dr Akao has received grants from the Japan Agency for Medical Research and Development; personal fees from Bristol Myers Squibb and Nippon Boehringer Ingelheim; and grants and personal fees from Bayer Yakuhin and Daiichi Sankyo. Dr Ako has received personal fees from Bayer Yakuhin and Sanofi; and grants and personal fees from Daiichi Sankyo, Dr Matoba has received grants from the Japan Cardiovascular Research Foundation; and personal fees from Nippon Boehringer Ingelheim, Daiichi Sankyo, AstraZeneca, and Bayer Yakuhin. Dr Nakamura has received grants and personal fees from Bayer Yakuhin, Daiichi Sankyo, and Sanofi; and personal fees from Bristol Myers Squibb and Nippon Boehringer Ingelheim. Dr Miyauchi has received personal fees from Amgen Astellas BioPharma, Astellas Pharma, Merck Sharp and Dohme, Bayer Yakuhin, Sanofi, Takeda Pharmaceutical, Daiichi Sankyo, Nippon Boehringer Ingelheim, and Bristol Myers Squibb. Dr Hagiwara has received grants and personal fees from Bayer Yakuhin and Nippon Boehringer Ingelheim; and personal fees from Bristol Myers Squibb. Dr Kimura has received grants from the Japan Cardiovascular Research Foundation grants and personal fees from Bayer Yakuhin, Daiichi Sankyo, Sanofi, Merck Sharp and Dohme, and AstraZeneca; and personal fees from Bristol Myers Squibb and Nippon Boehringer Ingelheim. Dr Hirayama has received grants and personal fees from Boston Scientific Japan, Otsuka Pharmaceutical, Sanofi, Astellas Pharma, Bristol Myers Squibb, Daiichi Sankyo, Bayer Yakuhin, Fukuda Denshi, Abbott Japan, Japan Lifeline, Takeda Pharmaceutical, and Sumitomo Dainippon Pharma; and personal fees from Toa Eiyo, Nippon Boehringer Ingelheim, Amgen Astellas BioPharma, and AstraZeneca. Dr Ogawa has received personal fees from Towa Pharmaceutical, Bristol Meyers Squibb, Pfizer, Toa Eiyo, Bayer Yakuhin, and Novartis Pharma. Dr Tsujita has received significant research grants from AMI, Bayer Yakuhin, Bristol Myers Squibb, EA Pharma, Mochida Pharmaceutical; scholarship funds from AMI, Bayer Yakuhin, Boehringer Ingelheim Japan, Chugai Pharmaceutical, Daiichi Sankyo, Edwards Lifesciences Corporation, Johnson and Johnson, Ono Pharmaceutical, Otsuka Pharmaceutical, and Takeda Pharmaceutical; and honoraria from Amgen, Bayer Yakuhin, Daiichi Sankyo, Kowa Pharmaceutical, Novartis Pharma, Otsuka Pharmaceutical, and Pfizer Japan; and belongs to the endowed departments donated by Abbott Japan, Boston Scientific Japan, Fides-one, GM Medical, ITI, Kaneka Medix, Nipro Corporation, Terumo, Abbott Medical, Cardinal Health Japan, Fukuda Denshi, Japan Lifeline, Medical Appliance, and Medtronic Japan. All other authors have reported that they have no relationships relevant to the contents of this paper to disclose.
